# Prevention of infectious tick-borne diseases in humans: Comparative studies of the repellency of different dodecanoic acid-formulations against *Ixodes ricinus *ticks (Acari: Ixodidae)

**DOI:** 10.1186/1756-3305-1-8

**Published:** 2008-04-08

**Authors:** Ulrich Schwantes, Hans Dautel, Gerd Jung

**Affiliations:** 1 Dr. R. Pfleger GmbH, 96045 Bamberg, Germany; 2 IS Insect Services GmbH, Haderslebener Strasse 9, 12163 Berlin, Germany

## Abstract

**Background:**

Ticks of the species *Ixodes ricinus *are the main vectors of Lyme Borreliosis and Tick-borne Encephalitis – two rapidly emerging diseases in Europe. Repellents provide a practical means of protection against tick bites and can therefore minimize the transmission of tick-borne diseases. We developed and tested seven different dodecanoic acid (DDA)-formulations for their efficacy in repelling host-seeking nymphs of *I. ricinus *by laboratory screening. The ultimately selected formulation was then used for comparative investigations of commercially available tick repellents in humans.

**Methods:**

Laboratory screening tests were performed using the Moving-object (MO) bioassay. All test formulations contained 10% of the naturally occurring active substance DDA and differed only in terms of the quantitative and qualitative composition of inactive ingredients and fragrances. The test procedure used in the human bioassays is a modification of an assay described by the U.S. Environmental Protection Agency and recommended for regulatory affairs. Repellency was computed using the equation: R = 100 - N_R_/N × 100, where N_R _is the number of non-repelled ticks, and N is the respective number of control ticks. All investigations were conducted in a controlled laboratory environment offering standardized test conditions.

**Results:**

All test formulations strongly repelled nymphs of *I. ricinus *(100-81% protection) as shown by the MO-bioassay. The majority of ticks dropped off the treated surface of the heated rotating drum that served as the attractant (1 mg/cm^2 ^repellent applied). The 10% DDA-based formulation, that produced the best results in laboratory screening, was as effective as the coconut oil-based reference product. The mean protection time of both preparations was generally similar and averaged 8 hours.

Repellency investigations in humans showed that the most effective 10% DDA-based formulation (~1.67 mg/cm^2 ^applied) strongly avoided the attachment of *I. ricinus *nymphs and adults for at least 6 hours. The test repellent always provided protection (83-63%) against *I. ricinus *nymphs equivalent to the natural coconut oil based reference product and a better protection (88-75%) against adult ticks than the synthetic Icaridin-containing reference repellent.

**Conclusion:**

We found that the 10% DDA-based formulation (ContraZeck^®^) is an easily applied and very effective natural repellent against I. ricinus ticks. By reducing the human-vector contact the product minimises the risk of transmission of tick-borne diseases in humans.

## Background

The most important and commonly found tick in northern Europe is *Ixodes ricinus *(L.), which acts as a vector of e.g. several *Borrelia *species causing Lyme Borreliosis, and of the **T**ick-**b**orne **E**ncephalitis (TBE) virus [1].

Tick-borne encephalitis is an endemic disease that is generally limited to certain geographic foci [2,3]. Up to 5% of ticks carry the TBE virus in endemic areas of Germany [2]. Thirty per cent of humans bitten by an infected tick develop symptoms of TBE (headache, fever, nausea) [1,2]. In most cases (90%), the symptoms disappear within about one week [2]. After an incubation period of up to 20 days, the remaining 10% of cases proceed to the second stage, which is characterised by the development of a severe form of meningo-encephalitis associated with the risk of constant neurological disorders (e.g., paraesthesia) [1]. A fatal outcome is reported in 1–2% of cases [1,2]. Vaccination against TBE is recommended as a preventive measure for individuals travelling to or living in risk areas. Currently, no drug treatment is available for TBE.

Unlike TBE, Lyme Borreliosis is widespread in Europe and temperate regions of the northern hemisphere [4,5] and infected ticks can be found at almost all locations, even in inner city parks [6]. Roughly 5 to 35% of ticks carry bacteria of the genus *Borrelia *(e.g., *B. burgdorferi*) [4,7]. The prevalence rate varies with the stage of tick development: approximately 20% of adult ticks, 10% of nymphs, and 1% of larvae are infected [7]. When bitten, 20–30% of humans show a seroconversion, 1.5–6% an infection, but only 0.3–4% actually develop Lyme Borreliosis [1,7]. Lyme Borreliosis is a multiform and multisystemic disease rapidly emerging over the last years [4]. In 40–60% of infected humans, the first manifestation is a spreading red rash around the bite (erythema migrans), which develops within a few days to weeks of the tick bite. If left untreated, meningopolyneuritis, myocarditis or arthritis can occur [4,7]. Lyme Borreliosis is treated with antibiotics.

*I. ricinus *ticks are restricted to habitats providing high relative humidities that do not fall below 80% for extended periods [8,9]. For host finding, the tick predominantly adopts the so-called ambush strategy, waiting for a host on a vantage point, e.g. grass or shrubs [10]. When a vertebrate animal or a human passes by, the tick quickly clings to the host, then, on the host, searches for an appropriate feeding site and starts feeding. Olfactory, visual and thermal receptors may play different roles in host and feeding site identification [11-13]. While feeding, *I. ricinus *secretes a variety of saliva components with e.g. anticoagulant, antiinflammatory, and immunosupressive action. These can directly damage the host and favour transmission and establishment of pathogens [14-18]. An increased risk of disease transmission with increased attachment time has been clearly demonstrated, e.g. for *B. burgdorferi *[10,19]. Since certain tick-borne pathogens, like the TBE virus, are transmitted to the host during the initial minutes of tick feeding [20], it is important to prevent tick bites completely.

Any person entering the vector's habitat whether for working or for leisure time activities is at risk. Protection from tick bites is best achieved by avoiding infested habitats, wearing protective clothing, and using tick repellent [4,21]. A repellent is defined by the Environmental Protection Agency of the United States as a 'pesticide product that causes insects to be driven or kept away from an identified area' [22]. In a broader and widely used sense, a repellent is a product intended to reduce the rate of biting from blood-sucking arthropods [23]. Given that a single bite from an infected arthropod can result in transmission of disease, a tick repellent must be able to prevent ticks from attaching to the skin. Unlike insecticides, repellents usually do not kill but rather prevent contact between the arthropod and the host and, as a consequence, can minimise the risk of acquiring tick-borne infections [4,24]. An ideal repellent should be effective, easy to apply and non-toxic to vertebrates, especially humans.

For use on skin, products containing *N, N*-diethyl-3-methylbenzamide (DEET) have been widely used for decades to protect against ticks and biting flies. Recently developed arthropod repellents, such as 1-methyl-propyl-2-(hydroxyethyl)-1-piperidinecarboxylate (picaridin), ethyl butylacetylaminopropionate (EBAAP), and (1*S*, 2'*S*)-2-methylpiperidinyl-3-cyclohexene-1-carboxamide (SS220) were also shown to effectively repel ticks/reduce the risk of tick bites [24-28]. However, concern about some serious toxic effects in humans and eco-toxicological problems associated with DEET-containing repellents has revived interest in plants as sources of natural-based repellents for protection [29-34].

The biocidal product (ContraZeck^®^) was developed and manufactured by Dr. R. Pfleger GmbH (Germany) to repel *I. ricinus *ticks. The active ingredient, dodecanoic acid (DDA), is a naturally occurring carboxylic acid that is the main acid in coconut oil and palm kernel oil, both of which are commonly used in foodstuffs. This saturated fatty acid is also a natural component of plant and animal tissues. The repellency of 10% DDA has been validated and patented [35]. We tested a variety of different topical 10% DDA-based formulations for their tick-repelling efficacy and tolerability during the process of ContraZeck^® ^research and development. The present paper describes the results of this laboratory screening process and presents the results of comparative repellency studies of the ultimately selected 10% DDA-based formulation, now available under the trademark ContraZeck^®^, versus reference products in humans.

## Methods

All tests were conducted by IS Insect Services GmbH in Berlin, Germany. The test methods have been described previously [36-38].

### Moving-object bioassay (laboratory screening)

#### Ticks

Laboratory screening tests were performed using unfed I. ricinus nymphs that were collected from different field sites in Berlin forest areas. All ticks were maintained at a shadowed outdoor site in glass vials within desiccators at a relative humidity of about 90%, natural temperature and photoperiod known to be suitable for the development and life cycle of I. ricinus [39]. Ticks were taken directly from the outdoor containers to the laboratory and acclimated to room temperature for about one or two hours before testing. Longer acclimation periods of up to two weeks were required in winter.

#### Test procedure

In the Moving-object (MO) bioassay, warmth and motion are used as attractants stimulating the natural tick behaviour of clinging to a passing host under controlled laboratory conditions. The apparatus used for these experiments was developed and described by Dautel et al. [36]. Briefly, a slowly rotating vertical drum is heated to a surface temperature of 35–37°C, which is regularly monitored by a remote infrared thermometer. A piece of filter paper (5 × 10 cm) fixed at an elevated position on the drum serves as the tick attachment site. Ticks attracted to the warmth approach the drum on a horizontally positioned glass rod that ends directly in front of the drum at a distance where the tick cannot reach the drum surface by its forelegs. As the drum rotates, however, the elevated surface of the drum covered by filter paper passes periodically by and the tick is able to cling to that surface and transfer to the drum. To test for repellency, the investigator applies a test substance to the filter paper and records whether or not the tick approaches and transfers to the drum and thereafter remains on the treated filter paper or drops off. The duration of each step of tick behaviour was measured to reveal more subtle repellent effects. The strength of this assay is that different compounds, formulations or products can be compared under standardised laboratory conditions, while confirming that the ticks under investigation are definitely in a natural host-seeking modus.

#### General test conditions

Prior to actual testing, a control run (blank test) was performed with 30 nymphal ticks without repellent but under otherwise identical test conditions. This control served to demonstrate sufficient activity of the ticks on the test day. The filter paper on the drum attachment site was then treated with one of the 10% DDA-based topical formulations or 10% DDA in ethanol (positive control). Each experimental run was carried out with 30 ticks, and each tick was only tested once. Since host-seeking behaviour is subject to temporal variation, experiments with control and test ticks were performed on the same day. Only active ticks that climbed voluntarily out of their glass vial after the investigator opened the vial were used for the tests. Using a fine brush, the ticks were placed individually on the glass rod with their anterior end facing the drum ≈ 1.5 cm away from the tip. All tests were performed under standardised conditions of room temperature (19–23°C), relative humidity (30–65%), and drum surface temperature (35.5–37.0°C).

##### Criteria assessed

*(1) Tick behaviour*. Each tick was graded for the following steps of host location:

a) Did the tick proceed to the drum? (YES/NO)

b) Did the tick attach to the treated or untreated elevated filter paper (attachment site)? (YES/NO)

c) Did the tick drop off the drum? (YES/NO)

*(2) Time course of tick activity*. The following times were recorded:

a) Time required for the tick to reach the tip of the glass rod, starting from the time it crossed a mark 1 cm from the tip.

b) Time required for the tick to climb from the tip of the glass rod to the elevated filter paper.

c) Time that the tick remained on the attachment site.

During each time interval a), b) and c), each tick was monitored for a maximum of 2 minutes. Ticks that did not move during the maximum test period were removed from the experiment and the time for this step was logged as 120 seconds.

#### Products tested

10% dodecanoic acid and seven 10% DDA-based topical formulations were investigated. All products tested were manufactured in compliance with the quality requirements of the current European Pharmacopeia (Ph. Eur.) in terms of identity, purity and content. 10% dodecanoic acid in ethanol was sprayed onto the elevated filter paper and the DDA-based formulations (lotions) were evenly applied to the filter paper with a roller. The paper was weighed before and after application of each test product to determine the applied dose. The MO-bioassay was performed using 5 × 10 cm strips of this filter paper. In each test, three such filter papers were assayed with 10 ticks each. The quantity of repellent applied per test was about 1 mg/cm^2^, corresponding to approximately 0.1 mg DDA/cm^2^. After this preliminary screening, the repellency of the 10% DDA-based formulation found to be most acceptable was compared to that of Zanzarin^® ^Bio-Hautschutz Lotion (Engelhard Arzneimittel, Germany), a commercially available, coconut fatty acid-based tick repellent.

#### Statistical analysis

The G-test [40] was used to test for differences in tick behaviour, such as the number of nymphs transferring to the rotating drum or dropping off. Statistical testing was conducted for differences between times required for specific behavioural steps using a one-way ANOVA and subsequent Student-Newman-Keuls test. *P*-values < 0.05 were regarded as significant. Data sets were analysed using the software package SPSS for Windows. The repellency of each test product was determined based on the number of ticks that: (1) did not approach the drum, (2) did not attach to the drum, and (3) dropped off the treated filter paper of the drum. Ticks fulfilling these criteria were classified as "repelled". Relative repellency (R) was computed using the equation R = 100-N_R_/Nx100, where N_R _is the number of non-repelled test ticks and N is the respective number of (non-repelled) control ticks.

### Tests in humans

Two small-scale trials in humans were then conducted to compare the repellent activity of the selected 10% DDA-based formulation (ContraZeck^®^)

*A*. to that of the natural coconut-oil based repellent Zanzarin^®^Bio-Hautschutz Lotion (Engelhard Arzneimittel, Germany) using *I. ricinus *nymphs

B. to that of the synthetic Icaridin-containing repellent Autan^® ^*Family *Zeckenschutz (Johnson Wax GmbH, Germany) using adult *I. ricinus*.

Disease-free, laboratory-reared ticks were used in all evaluations. The age of the nymphs in trial A was 5 months (n = 300) and 1.5 years (n = 700). The adult ticks in trial B were between 6 and 7 months (after moulting) old; their last blood meal was 8 to 9 months earlier (as nymphs). The ticks were maintained in a shadowed outdoor site within glass vials in desiccators at a relative humidity of 90%, normal temperature and photoperiod. One week before testing started, they were randomised and acclimated to room temperature at 90% relative humidity with a 16:8 h light:dark cycle.

For each study, a total of six volunteers (3 males and 3 females) aged 24 to 45 years (trial A) and 24 to 48 years (trial B) were tested in the controlled laboratory environment. During the tests the temperature was kept at a mean of 22.3 ± 1.2°C, a relative humidity of 58.9 ± 9.0%.

The test procedure used in the human bioassays was developed by Dautel [37]. It is a modification of an assay described by the U.S. Environmental Protection Agency and recommended for regulatory affairs for laboratory tests with volunteers [22]. The left and right lower leg of each volunteer was treated with one of the respective test products alternately on two different days. The surface area of each lower leg (test area) was calculated as the product of lower leg length (mean of two measurements of distance from the hollow of the knee to the heel lateral to the knee and on anterior aspect of each leg) and leg circumference (mean of 5 measurements taken at the ankle and knee and three equidistant points between). These measurements were used to calculate the amount of repellent applied per unit surface area (mg/cm^2^). The target dose of repellent, as recommended in the OPPTS 810.3700 product performance test guidelines of the U.S. Environmental Protection Agency, was 1.67 mg/cm^2 ^[22]. The amount of test product actually applied was determined by subtraction of before and after product weight measurements.

The volunteers were instructed to avoid coffee, tea and fragrance products on the day of testing. Immediately prior to treatment, each person washed the shaved leg with unscented (fragrance free) soap, rinsed it with water, cleaned it two times with a cloth soaked with 70% ethanol, rinsed the leg again with water and dried it with a towel. The volunteers applied and evenly distributed the weighed test product (~1.6 mg/cm^2^) to the entire test area using a gloved hand. White Vaseline was applied to the underside of an untreated copper disk (diameter: 3 cm, thickness: 0.1 mm), which was positioned in the centre of the treated skin area using forceps. With the aid of a stencil, a 13 cm diameter circle was marked around the copper disk. The ticks were applied to the lower legs (held in the vertical plane) of the seated volunteer. Thirty minutes after repellent application, two hungry ticks (nymphal (A) or adult (B) stages of *I. ricinus*) were placed on the untreated copper disk and observed for 5 minutes (maximum). The investigator recorded (1) whether the ticks crawled onto the treated skin or not, (2) whether the ticks dropped off the disk or skin, (3) whether the ticks crawled a distance of at least 5 cm (to the circle mark), and (4) how long it took the ticks to reach the mark. If they crossed the mark, the direction of motion (up, down, or horizontal) was also recorded in order to distinguish possible subtle repellent effects. Ticks that crawled onto the treated skin area and crossed the circle mark were classified as non-repelled, and those that did not as repelled.

At the start of each test, two ticks were placed together on the same disk, observed for 5 minutes, and then replaced with two new, untested ticks making a total of 12 ticks (adults: 6 female and 6 male ticks) per 30-minute cycle. Nymphs and adults were tested at times 30–60 min., 90–120 min., 150–180 min., 210–240 min., and 330–360 min after test product application.

Prior to product testing, a negative (untreated) control run with 12 ticks/volunteer was performed without repellent but under otherwise identical conditions. This control served to demonstrate sufficient activity of the ticks on the test day (criterion: at least 9 of 12 ticks crawled onto the skin and crossed the circle mark within 5 minutes).

#### Statistical analysis

The times required for ticks in the different groups to crawl onto the skin were tested for differences using a one-way ANOVA followed by the conservative Scheffé test. Statistical analyses were done using Statistika v. 8.0. Repellency of each product and differences between the efficacies of the two test products were evaluated by G-test [40] using the conservative Yate's correction in cases where samples with ≥ 30 ticks were compared. *P*-values < 0.05 were regarded as significant. Absolute repellency was expressed as the percentage of ticks that did not cross the circle mark on the treated skin (repelled ticks). Repellency (R) in relation to the control was computed using the aforementioned equation, where N_R _is the number of (non-repelled) ticks that crossed the circle mark and N is the respective number of (non-repelled) control ticks.

## Results

### Moving-object bioassay (laboratory screening)

To identify the most effective 10% DDA-based formulation, screening of 7 precursor preparations was performed using the MO-bioassay with a total of 720 ticks in five independent studies of comparable design.

#### Comparison of 10% DDA and the different DDA-based formulations

##### - Tick behaviour

The heated moving object (drum) proved to be a good attractant: ≥ 97% of the *I. ricinus *nymphs approached the drum in the absence of repellent. When the elevated filter paper on the drum (attachment site) was treated either with 10% DDA alone or with one of the DDA-based formulations, 100% and >93%, respectively, of the nymphs crawled to the tip of the glass rod (Table 1). Therefore, no distance effect was observed with DDA or any of the DDA-based formulations.

**Table 1:** Repellent effects of different DDA-based lotion-formulations against *I. ricinus *nymphs (MO-bioassay)

**Project**	**Crawling to the tip of the glass rod**		**Attaching the filter paper on the drum**		**Dropping off the attachment site**			**Repellency [%]**
	n	%	n	%	n	%	Significance*	
***Trial 1***								
Blank test	29	96.7	27	93.1	0	0		
10% DDS	30	100	28	93.3	25	89.3	*p *< 0.001	88.90
Batch 030138	30	100	23	76.7	22	95.7	*p *< 0.001	96.30
Batch 030142	29	96.7	20	69.0	15	75.0	*p *< 0.001	81.40
***Trial 2***								
Blank test	29/30	96.7	24/27	82.8/90.0	0/1	0/3.7		
10% DDS	30	100	24	80.0	21	87.5	*p *< 0.001	88.5
Batch 030174	30	100	20	66.7	17	85.0	*p *< 0.001	87.5
Batch 030175	29/29	96.7/96.7	21/20	72.4/69.0	19/17	90.5/85.0	*p *< 0.001	91.63/88.5
Batch 030176	28	93.3	22	78.6	20	90.9	*p *< 0.001	91.63
***Trial 3***								
Blank test	30	100	28	93.3	0	0		
10% DDS	30	100	24	80.0	19	79.2	*p *< 0.001	82.10
Batch 040088	30	100	23	76.7	19	82.6	*p *< 0.001	85.74
***Trial 4***								
Blank test	30	100	29	96.7	0	0		
Batch 040108	30	100	21	70.0	21	100	*p *< 0.001	100
Batch 050030	30	100	24	80.0	24	100	*p *< 0.001	100

The quantity of repellent applied per test was about 1 mg/cm^2^, corresponding to approximately 0.1 mg DDA/cm^2^. Batch No. 050030 is identical to the final product ContraZeck^® ^in terms of the qualitative and quantitative ingredients and fragrances. Batch No. 040088 and Batch No. 040108 are identical in terms of the qualitative and quantitative ingredients and fragrances. *Significance in relation to blank test

Fewer ticks clung to the elevated filter paper treated with either 10% DDA or any of the respective DDA-based formulations than to the untreated filter paper, but the percentage of nymphs that transferred to the drum attachment site was not significantly different from the controls. In addition, the nymphs that did not cling to the elevated filter paper showed no typical distance effect in response to the repellent. Most of them dropped off the glass rod even when they stretched their forelegs and touched the attachment site of the moving drum.

The 10% DDA and all of the DDA-based formulations significantly repelled those tick nymphs that transferred to the treated filter paper. Between 75 and 100% of the nymphs dropped off the treated filter paper within only a few seconds; this behaviour was significantly different from that of the controls.

All products tested achieved a relative repellency of 81.4 to 100 per cent. The repellency of 10% DDA (positive control) was 82.1% in one test and approximately 89% in the two others. One DDA-based formulation achieved a repellency of only 81.4%, but the other six formulations achieved repellency levels of 86 to 100 per cent. Accordingly, the repellency of the six DDA-based formulations was at least as high as that of 10% DDA.

##### - Time course of tick activity

**Figure 1:**
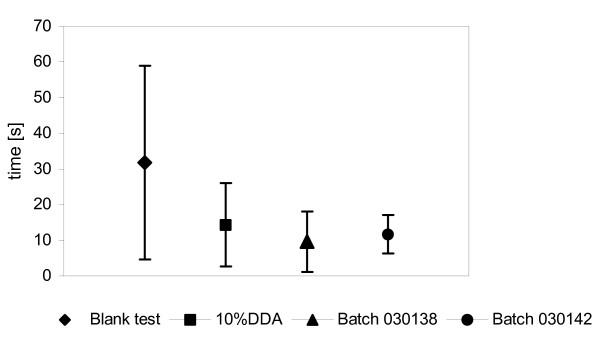
**MO-bioassay. Mean times (± S.D.) tick nymphs stayed attached to the filter paper**. The filter paper on the attachment site of the rotating drum was treated either with 10% DDA (n = 28) or with one of the 10% DDA-based formulations (Trial 1: batch no. 030138, n = 23; batch no. 030142, n = 20). The control run (blank test, n = 27) was performed without repellent but under otherwise identical conditions. Differences between the control and each of the test substances are statistically significant (ANOVA, *P *< 0.001).

The mean times required to reach the tip of the glass rod and to transfer to the rotating drum did not differ significantly between the control and test groups (ANOVA, *P *> 0.05). Therefore, no distance effect associated with repellent exposure was observed. The tick nymphs stayed attached to filter paper treated with either 10% DDA or one of the DDA-based formulations for significantly less time than on untreated filter paper (ANOVA, *P *< 0.001). Most ticks dropped off the treated area within a few seconds. In addition, the nymphs remained on filter paper treated with the respective DDA-based formulations for significantly shorter periods than on filter paper treated with 10% DDA (Figure 1).

#### Repellency of the selected 10% DDA-based formulation versus Zanzarin^®^

The repellency of the 10% DDA-based formulation that produced the best results in screening was compared to that of the reference product Zanzarin^® ^in a further study using the MO-bioassay. The course of repellency over time was observed in 210 tick nymphs for a total of 8 hours after application of the respective test substance (Table 2). The DDA-based formulation exhibited maximum relative repellency (100%) 1, 4 and 8 hours after application. Repellency of the reference product was slightly lower after 1 hour (96.5%) and also varied over time. However, both the DDA-based formulation and the reference product proved to be equally repellent to *I. ricinus *nymphs over 8 hours. Ticks spent significantly less time on filter paper treated with either of the two repellents than on untreated filter paper. The two repellent formulations did not exhibit any significant differences in regard to the time course of tick activity.

**Table 2:** Repellent effects of ContraZeck^® ^and Zanzarin^® ^against *I. ricinus *nymphs over time (MO-bioassay)

**Test formulation**	**Crawling to the tip of the glass rod**		**Attaching the filter paper on the drum**		**Dropping off the attachment site**			**Repellency [%]**
	n	%	n	%	n	%	Significance	
Blank test	30	100	28	93.3	0	0		
DDA-based form. Batch 040088 – 1 h	30	100	23	75.7	23	100	*p *< 0.001* *p *> 0.05**	100
Batch 040088 – 4 h	30	100	25	83.3	25	100	*p *< 0.001* *p *< 0.05**	100
Batch 040088 – 8 h	30	100	23	75.7	23	100	*p *< 0.001* *p *> 0.05**	100
Reference^# ^– 1 h	28	93.3	23	82.1	22	95.7	*p *< 0.001*	96.5
Reference^# ^– 4 h	30	100	29	96.7	24	82.8	*p *< 0.001*	82.1
Reference^# ^– 8 h	30	100	25	83.3	21	84.0	*p *< 0.001*	85.7

The quantity of the DDA-based formulation (ContraZeck^®^) applied per test was about 1 mg/cm^2^, corresponding to approximately 0.1 mg DDA/cm^2^. ^# ^The reference product Zanzarin^® ^(Bio-Hautschutz Lotion) was applied per test in a concentration of 1.05 mg/cm^2^.

Significance: *in relation to blank test, **in relation to Zanzarin^®^. Differences in repellency between both products are significant only 4 hours after application.

### Tests in humans

#### Trial A: Repellency of ContraZeck^® ^versus Zanzarin^® ^against I. ricinus nymphs

*I. ricinus *nymphs were strongly repelled by both the selected 10% DDA-based formulation and the reference product Zanzarin^® ^for up to 6 hours after application (G-values always >38.5, *P always *< 0.001). Quantitative differences in the repellency of the two products between volunteers were observed. However, both of the two products exhibited statistically significant repellency in all volunteers and at each sampling time.

**Figure 2:**
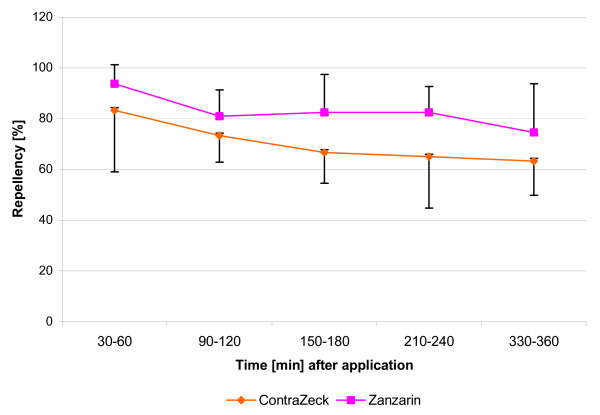
**Test in humans [A]. Relative repellency of ContraZeck^® ^and Zanzarin^® ^against *I. ricinus *nymphs**. Each data point in the graph is the mean ± S.D. of relative repellency (with respect to the controls) in six volunteers and of 12 nymphys tested in each volunteer (n = 72) at the respective time interval.

Figure 2 shows the mean repellency of both products in tick nymphs in relation to the respective controls over the whole observation time of 360 minutes after application. Whereas more than 94% of all control nymphs crawled onto the untreated skin, both repellents prevented more than 83% (ContraZeck^®^) and 94% (Zanzarin^®^) of them from crossing the 5 cm circle mark during the first observation cycle (30–60 min after application). Most of the nymphs dropped already off the copper disk, a small number only dropped off the treated skin. No significant differences in mean repellency were found between the two products at any time. Both the test formulation and reference product remained as effective as in the preliminary experiments.

The percentage of tick nymphs repelled decreased slowly and moderately over time (up to 6 h), but the repellency of the test and reference product was always significantly different from that of the controls. At 6 h, relative repellency of the DDA-based formulation was 63% in *I. ricinus *nymphs compared to 75% for the reference product (difference not significant).

The test and reference products did not exhibit any differences with respect to the time required for the ticks to crawl onto the treated skin or in the direction of their walk. Control ticks exhibited a strong tendency to crawl upwards on untreated skin. In contrast, test ticks walking on treated skin preferred to crawl downwards to escape the host [37]. A mean 62% of control ticks crawled up and 15% down (the others to the side), whereas only 2 nymphs per test substance crawled up, but 80% (ContraZeck^®^: G = 99.5, *P *< 0.001) and 50% (Zanzarin^®^: G = 69.8, *P *< 0.001) crawled downwards on the treated skin. In this respect, there were no significant differences between the test and reference product.

#### Trial B: Repellency of ContraZeck^® ^versus Autan^® ^Family against adult ticks of I. ricinus

**Figure 3:**
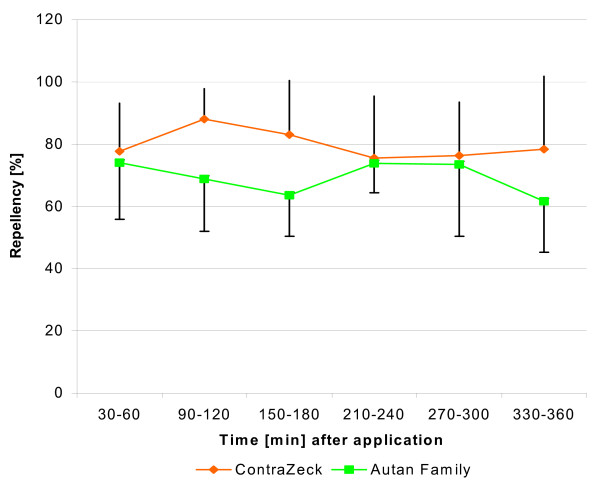
**Test in humans [B]. Relative repellency of ContraZeck^® ^and Autan^® ^*Family *against adult *I. ricinus *ticks**. Each data point in the graph is the mean ± S.D. of relative repellency (with respect to the controls) in six volunteers and of 12 adult ticks tested in each volunteer (n = 72) at the respective time interval.

Of the two products tested, that containing 10% DDA provided the most effective protection (relative repellency: 88-75.5%). There was an overall statistically significant tendency in favour of the 10% DDA-based formulation (ContraZeck^®^) when compared with the synthetic Icaridin-containing product (Autan^® ^*Family*) over all volunteers and all time points (G = 12.4, *P *< 0.01). Compared to the controls, both the test product and the reference product provided statistically significant and long-lasting protection (88-62%) in all volunteers and at each time point for up to 6 hours after application (G-values always >61.9, *P *always < 0.001) (Figure 3). In terms of relative repellency, both products were quite similar at 1, 4 and 5 hours after application, whereas there was a statistically significant difference in repellency between ContraZeck^® ^and Autan^® ^*Family *at 2, 3 and 6 hours (2 h: G = 6.4 (*P *< 0.05); 3 h: G = 6.4 (*P *< 0.05); 6 h: G = 4.2 (*P *< 0.05).

Differences in repellent reaction between male and female ticks were not observed. There were almost no further differences between both products regarding the number of ticks entering the treated skin or between the walking directions of such ticks that were not repelled. However, both products induced more ticks to walk down than in the controls.

## Discussion

The European Parliament classifies substances as "repellents and attractants" (biocidal product-type 19) that are "used to control harmful organisms (invertebrates, vertebrates), by repelling or attracting, including those that are used for human or veterinary hygiene either directly or indirectly" [41]. Dodecanoic acid (DDA), the active substance in the tick repellent ContraZeck^®^, is intended for use on human skin. When applied as a repellent, DDA therefore indirectly serves as a human hygiene product used as a preventive measure. Consequently, the respective DDA-formulation must be classified as a biocidal product type 19 according to Annex V of Directive 98/8/EC [41]. The repellent must therefore fulfil several requirements to be accepted by regulatory authorities and users. The results of the presented investigations demonstrate convincingly that the DDA-based formulation meets these requirements.

### Protection from tick bites

#### Moving-object bioassay

As clearly demonstrated by laboratory screening, each of the seven DDA-based formulations tested significantly reduced the proportion of *I. ricinus *nymphs attaching to repellent-treated filter paper on the rotating drum of the MO-bioassay apparatus. Although a distance effect covering a few mm or more can be excluded, a small number of ticks dropped off in the phase of clinging to the drum surface. The majority of ticks, however, dropped off the treated filter paper within seconds, a behaviour rarely observed in the controls. This demonstrates a clear repellent effect.

In all experiments, it was clear that the nymphs displayed their natural host-seeking behaviour during the tests. Arthropods engaged in host seeking are more difficult to repel than individuals tested in the absence of any attractive stimuli [42]. This was shown for ticks using the MO-bioassay, since DEET could repel *I. ricinus *nymphs even from a short distance when there was no attractant available, whereas a high proportion of nymphs initially walked onto the DEET-treated filter paper of the rotating drum that attracted the ticks by warmth [36]. Thus, the MO-bioassay can assess more aspects of repellency than an assay lacking such an attractive component. This is a major advantage since repellents used for personal protection must effectively counteract all the attractive effects of a potential host [38]. Therefore, the MO-bioassay is considered a suitable laboratory test system for potential tick repellents. The good prospective quality of the MO-bioassay is further shown by the results of the human studies that differed only slightly from those of the laboratory screening.

The fact that most of the nymphs dropped off the treated filter paper suggests that the tested DDA-based formulations may act as contact repellents. Such contact repellency was proven for the pyrethroid permethrin [43], a sodium channel blocker that did not repel ticks in the gas phase [26]. One can suspect that the different DDA-based formulations either had irritant properties that warded off the ticks and/or other effects that modified the surface properties of the treated area. However, from the MO-bioassay results it cannot be ruled out that DDA might nevertheless ultimately act in the gas phase at concentrations that are only high enough immediately above the evaporating surface at 35°C, a temperature close to the melting point of the substance.

However, irrespective of its mode of action, the proven repellency of each of the 10% DDA-based formulations unequivocally demonstrates that this concentration is adequate for effective protection against tick bites. The test results also showed that the selected fragrances and inactive ingredients of each product formulation did not relevantly influence the repellent effect of the active principle, dodecanoic acid.

The efficacy of the selected DDA-based formulation was comparable to that of the reference product Zanzarin^® ^(Bio-Hautschutz Lotion), which had already been independently verified as being very effective against hard ticks [44]. In these previous investigations, the coconut oil-based product Zanzarin^® ^was identified as the most effective product currently available on the market.

#### Tests in humans

The repellency of the 10% DDA-based formulation found to be the most acceptable in laboratory screening was additionally compared to that of two reference products in humans. Under the described standardised test conditions [38], nymphal and adult life stages of *I. ricinus *were strongly deterred from crossing uncovered skin treated with either the test or one of the reference products for at least six hours. Hereby, the DDA-based preparation was found to be just as effective as the natural reference product Zanzarin^® ^in repelling *I. ricinus *nymphs. Comparable experimental conditions were used to test eight other commercially available repellents against ticks of the species *I. ricinus *in a previous study with volunteers [37]. Two products prevented more than 90% of the nymphs from crawling a distance of 5 cm over treated skin (arm or leg) during the observation period of 15 to 45 min after repellent application. The other six products either repelled lower proportions of the ticks (70–80%) or showed no repellent effect at all (<50%). Accordingly, both the DDA-based formulation and the reference product used in the present human study showed comparable efficacy against *I. ricinus *nymphs as the most effective repellents in the previous study.

The selected DDA-based formulation ContraZeck^® ^was also proved to be highly effective against adult ticks of *I. ricinus*. Overall, there was a statistically significant tendency in favour of ContraZeck^® ^when compared with the synthetic reference product Autan^® ^*Family *over all volunteers and all time points.

The present tests in humans showed, that ticks need not necessarily come into direct contact with the tested formulation in order to be repelled by them as the great majority of the ticks dropped off the copper disk before they had come in contact with the treated skin.

Another criterion, albeit for a more subtle repellent effect, may be the direction the tick crawls on treated skin. Once on a host, *I. ricinus *nymphs and adults normally prefer to crawl upwards when seeking a feeding site. In the present assays, however, most of the ticks that crossed repellent-treated skin walked downwards, irrespective of the type of repellent used. This finding may signify that such ticks that were not classified "repelled" according to test criteria might nevertheless have had the motivation to walk off the host.

Theoretically, field tests with volunteers are the preferable method for evaluation of repellents for human use [42], but they are presumably unethical in Europe because they pose the risk of transmission of tick-borne diseases. Furthermore, conducting such studies indoors makes it possible to reduce potential confounding variables, such as density of tick population, the level of the ticks' hunger, temperature, humidity, and the wind speed that can make it difficult to analyse comparisons among products made in outdoor-field trials [38]. Field tests require therefore high numbers of test replications. The volunteer studies used here, in contrast, allow direct observation and quantification of tick behaviour under more standardised conditions, including minimal risk of volunteers to acquire tick-borne diseases. The ratio for using this specific test in favour of the EPA procedure is discussed in Dautel [37].

The observed inter-individual differences in repellency are quite normal considering that only a small number of volunteers were tested and that the attractiveness of humans to other blood-sucking arthropods also varies [45,46].

In conclusion, the DDA-based formulation convincingly meets the requirements of an effective repellent and prevents ticks from piercing exposed skin. This can be achieved either by preventing the tick from clinging to the body at all or by inducing the tick to drop off once it comes in contact with treated skin.

### Duration of repellency

The investigations showed that the tested DDA-based formulations provided significant tick bite protection for up to 6 hours in humans and up to 8 hours in the laboratory-testing device. The mean protection times of ContraZeck^® ^and the reference products against bites of *I. ricinus *nymphs and adults were comparable. Under field conditions, however, the degree of protection can be influenced by several factors, including environmental ones, like habitat structure, weather, tick activity/density, and host-typical properties like the degree of human exercise, the kind of clothes or fragrances used, or the quantity of repellent applied [24,37].

### Tolerance

No toxic or allergic reactions to dodecanoic acid have been reported, and DDA was shown to be safe when used on skin. The Cosmetic Ingredient Review [47] concluded that DDA is safe in cosmetic use up to a concentration of 25%.

## Conclusion

We conclude that the DDA-based formulation ContraZeck^® ^is an easily applied and effective repellent against nymphal and adult life stages of I. ricinus. The product can be expected to provide a safe as well as a long-lasting repellent effect under circumstances in which it is crucial to be protected against tick bites that might transmit disease.

## Competing interests

The authors declare that they have no competing interests. Funding is acknowledged in the article.

## Authors' contributions

US conceived the studies, and participated in their design and coordination. GJ developed, analysed and manufactured the different DDA-based formulations. HD carried out the laboratory screening tests and human bioassays, and performed the statistical analysis. US and HD reviewed and provided input into the first draft of this manuscript. US and HD take responsibility for the interpretation of the results and for critical revision of the manuscript. All authors read and approved the final manuscript.
